# Untargeted Metabolomics Analysis Reveals Differential Accumulation of Flavonoids Between Yellow-Seeded and Black-Seeded Rapeseed Varieties

**DOI:** 10.3390/plants14050753

**Published:** 2025-03-01

**Authors:** Shulin Shen, Yunshan Tang, Daiqin Liu, Lulu Chen, Yi Zhang, Kaijie Ye, Fujun Sun, Xingzhi Wei, Hai Du, Huiyan Zhao, Jiana Li, Cunmin Qu, Nengwen Yin

**Affiliations:** 1 Integrative Science Center of Germplasm Creation in Western China (CHONGQING) Science City, College of Agronomy and Biotechnology, Southwest University, Chongqing 400715, China; ssl7159@email.swu.edu.cn (S.S.); ljn1950@swu.edu.cn (J.L.); 2 Academy of Agricultural Sciences, Southwest University, Chongqing 400715, China

**Keywords:** yellow-seeded rapeseed, black-seeded rapeseed, untargeted metabolomics, seed development, flavonoids, molecular networking

## Abstract

Rapeseed (*Brassica napus*) is an important oilseed crop and yellow-seeded and black-seeded varieties have different metabolite profiles, which determines the quality and edibility of their oil. In this study, we performed a non-targeted metabolomics analysis of seeds from four rapeseed varieties at eight developmental stages. This analysis identified 4540 features, of which 366 were annotated as known metabolites. The content of these metabolites was closely related to seed developmental stage, with the critical period for seed metabolite accumulation being between 10 and 20 days after pollination. Through a comparative analysis, we identified 18 differentially abundant flavonoid features between yellow-seeded and black-seeded rapeseed varieties. By combining the flavonoid data with transcriptome data, we constructed a gene regulatory network that may reflect the accumulation of differentially abundant flavonoid features. Finally, we predicted 38 unknown features as being flavonoid features through molecular networking. These results provide valuable metabolomics information for the breeding of yellow-seeded rapeseed varieties.

## 1. Introduction

Plants have developed specialized metabolic pathways throughout their evolution that enable them to adapt to various environments [1,2]. Together, these metabolic pathways produce about 200,000 to 1 million abundant secondary metabolites in plants [3]. These metabolites can be divided into (1) N-containing compounds, (2) phenylpropanoids, (3) benzenoids, (4) flavonoids, and (5) terpenes [4]. The levels of these metabolites and their derived products are closely related to the flavors [5,6], yield [7,8], and stress resistance [9,10] of plants. Moreover, marked differences have appeared in the metabolic profiles of domesticated crops compared to their wild relatives [11].

In plants, the main substances involved in color formation are the chlorophylls, carotenoids, betalains, and flavonoids. The formation of color in rapeseed seeds is primarily due to flavonoids. The biosynthesis of flavonoid biosynthetic pathways starts with malonyl-coenzyme A (CoA), produced through the polyketide pathway, and 4-coumaroyl-CoA, produced through the shikimate-phenylpropanoid pathway [12,13]. These two precursors combine to form naringenin chalcone, the first metabolite of the flavonoid pathway, in a reaction catalyzed by chalcone synthase (CHS). Naringenin chalcone is successively converted by chalcone isomerase (CHI), flavanone-3-hydroxylase (F3H), flavonoid 3′-hydroxylase (F3′H), dihydroflavonol 4-reductase (DFR), leucoanthocyanidin dioxygenase (LDOX), and anthocyanidin reductase (ANR) into naringenin, dihydrokaempferol, dihydroquercetin, leucocyanidin, cyanidin, and epicatechin, respectively [14]. These proanthocyanidin precursors are transported from the endoplasmic reticulum to the vacuole through three modes: via vesicle transport, via glutathione S-transferase (GST), and via membrane transporters [15]. In the vacuole, proanthocyanidin precursors undergo polymerization and oxidation, ultimately turning the seeds brown or black [16]. In addition, the dihydrokaempferol and dihydroquercetin products formed by the above reactions can be used as substrate by flavonol synthase (FLS) to produce kaempferol and quercetin, which can themselves form diverse flavonoids through modifications such as glycosylation and methylation [17]. The modification pathways of these flavonoid metabolites have not yet been well characterized.

Rapeseed (*Brassica napus* L.) is an important source of edible oil, accounting for approximately 13% of global edible oil production [18]. Yellow-seeded rapeseed varieties are highly sought after by breeders owing to their thin seed coat and high oil content [19,20]. The establishment of rapeseed seed coat color is related to the accumulation of proanthocyanidins, lignin, and melanin [21]. Among these pigments, proanthocyanidins produced by the flavonoid pathway are the main substances that determine the pigmentation of the rapeseed seed coat [22].

Recent studies have shown that knocking out genes homologous to those in the *Arabidopsis thaliana* flavonoid biosynthetic pathway, such as *TRANSPARENT TESTA 8* (*BnTT8*), *BnTT2*, or *BnTT12*, in black-seeded rapeseed varieties can produce rapeseed plants with yellow seeds and higher oil content [19,23,24]. This increase in oil content is due to the decreased overall activity of the flavonoid pathway, thus redirecting the flow of its substrate malonyl-CoA to the oil biosynthesis pathway, thereby increasing seed oil content [17,25].

Lignin and flavonoids are produced downstream of the phenylpropanoid pathway, making the relationship between lignin and the pigments responsible for rapeseed seed coat color more complicated; nonetheless, rapeseed varieties with yellow seed coats have lower lignin content [26]. However, because the lignin pathway competes with the flavonoid pathway for 4-coumaroyl CoA, rapeseed seeds can also become yellow when overexpressing the key gene *CINNAMOYL COA REDUCTASE* (*BnCCR*) for lignin biosynthesis [27].

Compared to the previous two pigments, melanin has been less reported, possibly because the concept of plant melanin remains controversial, together with errors in the melanin extraction methods used in previous studies [21]. For example, recent studies in barley (*Hordeum vulgare*) and rice (*Oryza sativa*) suggest that flavonoids may form melanin through oxidation [28], but their role in rapeseed has yet to be studied. Overall, the competition among the metabolic pathways producing proanthocyanidins, lignin, and fatty acids is a fundamental reason for the formation of different colors and qualities of rapeseed seeds.

In recent years, the metabolic changes in seeds throughout their development have received more attention. For example, the analysis of metabolism at all developmental stages in rice [29], tomato (*Solanum lycopersicum*) [30], and kiwi fruit (*Actinidia chinensis*) [31,32] has helped us to better understand the mechanisms affecting fruit quality. Here, we used an untargeted metabolomics approach to explore the metabolite differences among four rapeseed varieties during eight stages of seed development. We detected differences in the dynamic accumulation of flavonoid features between yellow-seeded and black-seeded rapeseed varieties during seed development. Combining transcriptome and metabolome data, we identified a gene regulatory network that may affect the accumulation of flavonoid compounds. We also predicted unknown features through molecular network analysis, enriching our understanding of the metabolic differences between yellow-seeded and black-seeded rapeseed varieties. In summary, this study comprehensively reveals the metabolic changes of rapeseed seeds during their development and provides new insights into the establishment of rapeseed seed coat pigmentation.

## 2. Results

### 2.1. Differences in Quality Traits Among Mature B. napus Seeds

**Figure 1:**
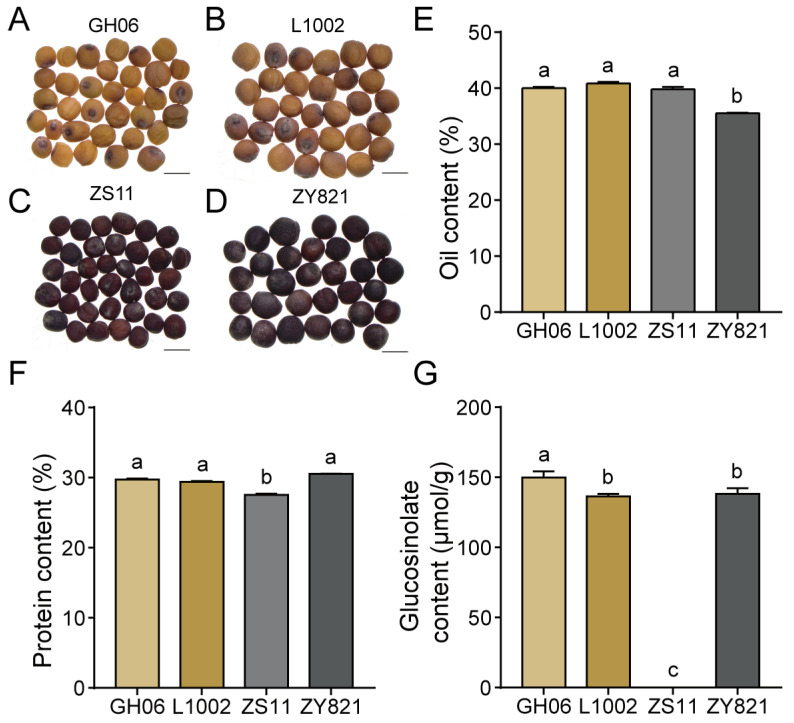
Analysis of seed composition from yellow-seeded and black-seeded *B. napus* cultivars. Representative photographs of seeds from the *B. napus* cultivars GH06 (**A**), L1002 (**B**), ZS11 (**C**), and ZY821 (**D**). In (**A**–**D**), the ruler in the lower right corner indicates 2 mm. Percentage of oil content (**E**), protein content (**F**), and glucosinolate content (in μmol/g) (**G**) in mature fresh-weight seeds from the *B. napus* cultivars GH06, L1002, ZS11, and ZY821. In (**E**–**G**), lowercase letters indicate significant differences, as determined by *t*-tests.

We used four *B. napus* varieties, the black-seeded varieties ZS11 and ZY821, and the yellow-seeded varieties GH06 and L1002, in this study (Figure 1A–D). We measured various quality traits from the mature seeds of these four varieties with a near-infrared spectrometer (NIRS DS2500 analyzer, Foss Analytical A/S). Of the four varieties, the two yellow-seeded *B. napus* varieties, GH06 and L1002, shared similar quality traits (Supplementary Data S1). The oil content of ZY821 was 35.5%, thus significantly lower than the oil content of the three other varieties, which was about 40% (Figure 1E). The protein content of ZS11 was 27.55%, which was significantly lower than that in the three other varieties, which was about 30% (Figure 1F). In addition, in agreement with the notion that ZS11 is a widely planted low-glucosinolate variety, mature ZS11 seeds only contained low levels of glucosinolate substances, amounting to 7.74 μmol/g seed fresh weight, which was much lower than in the three other varieties (Figure 1G). These results highlight the differences in quality traits of seeds from these four *B. napus* varieties.

### 2.2. Metabolite Profiling of B. napus Seeds

**Figure 2:**
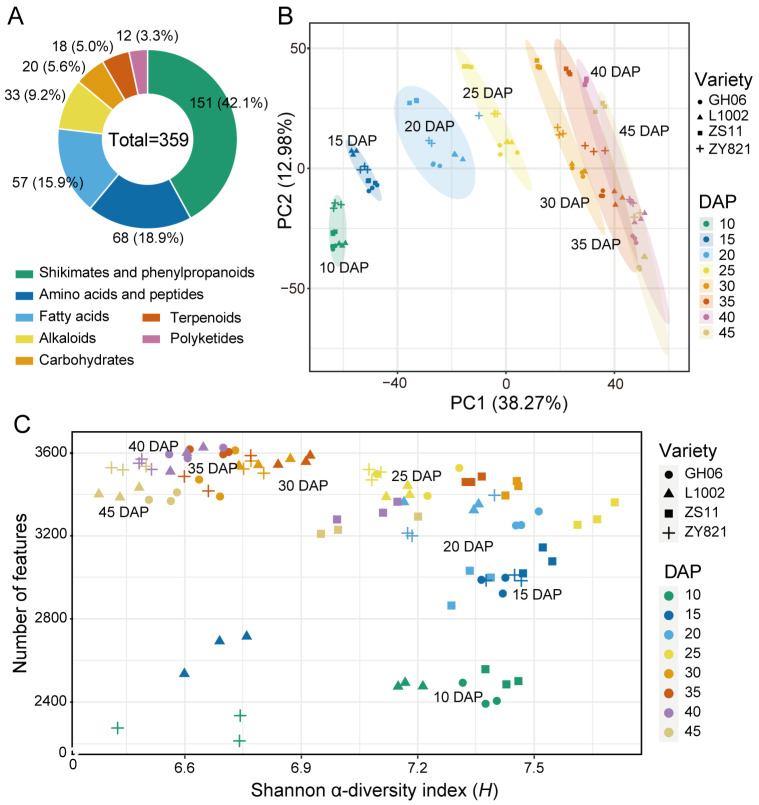
Metabolite analysis of *B. napus* varieties with yellow or black seeds at eight developmental stages. (**A**) Pie chart of the classification of 359 known metabolites. (**B**) Principal component analysis (PCA) of detected features from the seeds of four rapeseed varieties across seed development. (**C**) Scatterplot showing the relationship between the number of detected features and the Shannon *α*-diversity index (*H*) as an indicator of feature richness for each sample.

In this study, we performed a non-targeted metabolomics analysis of seeds from the four *B. napus* varieties at eight developmental stages (10, 15, 20, 25, 30, 35, 40, and 45 days after pollination [DAP]). We obtained high and positive correlations (r > 0.9) among quality control samples, indicative of the repeatability and reliability of the measured data (Figure S1). We detected a total of 4540 features, of which 366 were annotated as known metabolites. Of these 366 known metabolites, 359 could be classified into seven pathways by NPClassfire, representing 151 shikimate and phenylpropanoid compounds, 68 amino acids and peptides, 57 fatty acids, 33 alkaloid compounds, 20 carbohydrates, 18 terpenoid compounds, and 12 polyketide compounds (Figure 2A, Supplementary Data S2). We performed a principal component analysis (PCA) to explore the metabolic landscape across samples, which revealed an ordered distribution of clusters from seeds at 10 DAP to 45 DAP from left to right along principal component 1 (PC1) (Figure 2B). Notably, samples from the yellow-seeded and black-seed varieties at the same seed developmental stage were largely separated along PC2 (Figure 2B).

To contrast the patterns of feature diversity at different seed developmental stages, we calculated the α-diversity scores based on Shannon entropy (*H*). We then plotted the number of features as a function of the α-diversity index (*H*) for each sample in a scatter plot. The number of features for samples from the four varieties increased with seed development, while the α-diversity index (*H*) decreased after reaching a peak at 25 DAP (Figure 2C). We conclude that the diversity of metabolites in the early stages of seed development gradually decreases with seed maturity. Overall, the above metabolomic data reflect the characteristics of seed development time and seed color for the analyzed *B. napus* varieties.

### 2.3. Differences in Detected Flavonoid Features Between Yellow-Seeded and Black-Seeded B. napus Varieties

**Figure 3:**
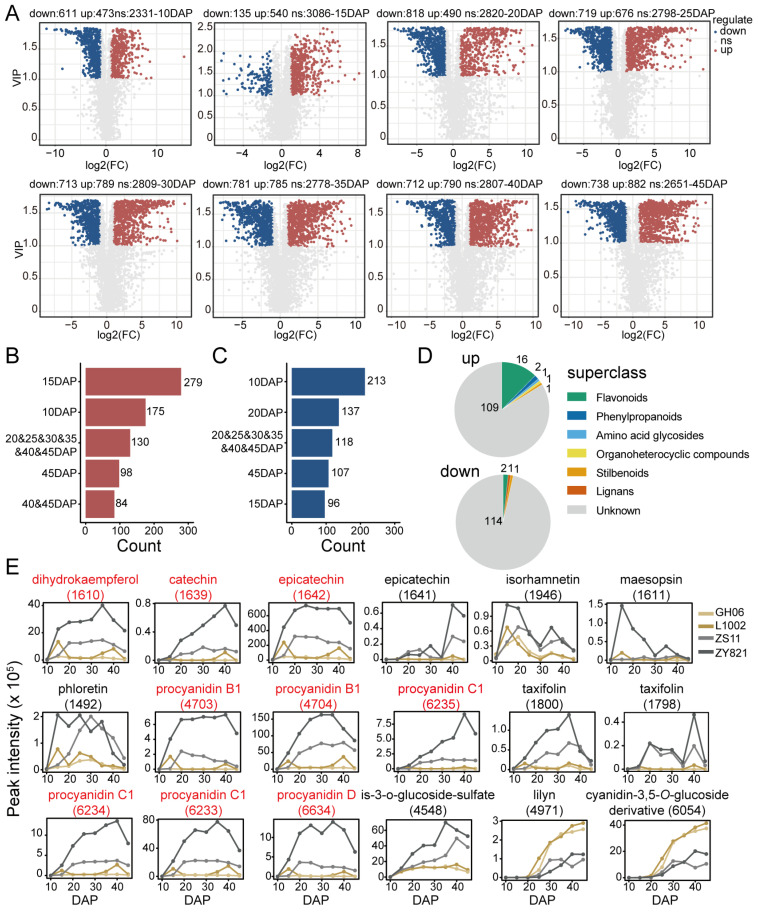
Analysis of differentially abundant features between *B. napus* varieties with yellow or black seeds. (**A**) Volcano plots showing the differentially abundant features between the seeds of yellow or black *B. napus* varieties at 10 days after pollination (DAP), 15 DAP, 20 DAP, 25 DAP, 30 DAP, 35 DAP, 40 DAP, and 45 DAP. VIP, variable importance in projection value. Bar chart of the top five intersections of up- (**B**) and downregulated (**C**) features for yellow-seeded rapeseed and dark-seeded rapeseed. (**D**) Classification of upregulated and downregulated features common from 20 DAP to 45 DAP. (**E**) Accumulation profiles of 18 differentially abundant features in four rapeseed varieties across seed development. Red titles indicate flavonoid features showing large differences in their contents between the two black-seeded varieties.

To investigate the differences between the yellow-seeded and dark-seeded *B. napus* varieties analyzed in this study, we used an orthogonal partial least-squares discriminant analysis (OPLS-DA) model to compare the metabolite features identified in the two yellow-seeded *B. napus* varieties and the two dark-seeded *B. napus* varieties at each seed developmental stage. We then selected the differentially abundant features for each comparison by using a variable importance in projection (VIP) value ≥ 1 in the OPLS-DA model and a fold-change (FC) above 2 (more abundant in the yellow-seeded varieties) or below 0.5 (less abundant in the yellow-seeded varieties). The differential features for each stage are shown in Figure 3A. Among all stages, 15 DAP had the least number of differential features (down: 135, up: 540). The numbers of features upregulated or downregulated in the other stages were mostly between about 600 and 800. We drew Upset diagrams to explore the distribution of these differentially abundant features across groups of *B. napus* varieties (based on seed color) and seed developmental stages (Figure S2). The Upset diagrams of upregulated and downregulated features indicated that four of the top five intersections are consistent, including differentially accumulated features unique to 10 DAP, 15 DAP, and 45 DAP and common from 20 DAP to 45 DAP (Figure 3B,C). Further analysis of the common differentially abundant features showed that three of these 118 downregulated common features were annotated, representing one stilbenoid, two flavonoids, and one lignan. Of the 130 upregulated features, 21 were annotated, comprising 16 flavonoid compounds, two phenylpropanoid compounds, one amino acid glycoside, one organoheterocyclic compound, and one stilbenoid compound (Figure 3D). These results suggest that the differentiation of the flavonoid metabolic pathway between yellow-seeded and black-seeded *B. napus* varieties starts at 20 DAP and remains differentiated until seed maturity.

The accumulation profiles of 18 differentially abundant flavonoid features are shown in Figure 3E (Supplementary Data S3). Of these, 16 metabolites accumulated to high levels in the black-seeded *B. napus* varieties before decreasing in the later stages of seed development. The remaining two metabolites showed an opposite profile, with increasing accumulation in yellow-seeded *B. napus* varieties over time. Interestingly, nine flavonoid features showed large differences in their contents between the two black-seeded *B. napus* varieties (labeled in red in Figure 3E). These results suggest that black-seeded *B. napus* varieties also exhibit major differences in their flavonoid metabolic pathway, which was not noticed in previous studies.

### 2.4. Differences in Flavonoid Features Between ZS11 and ZY821

**Figure 4:**
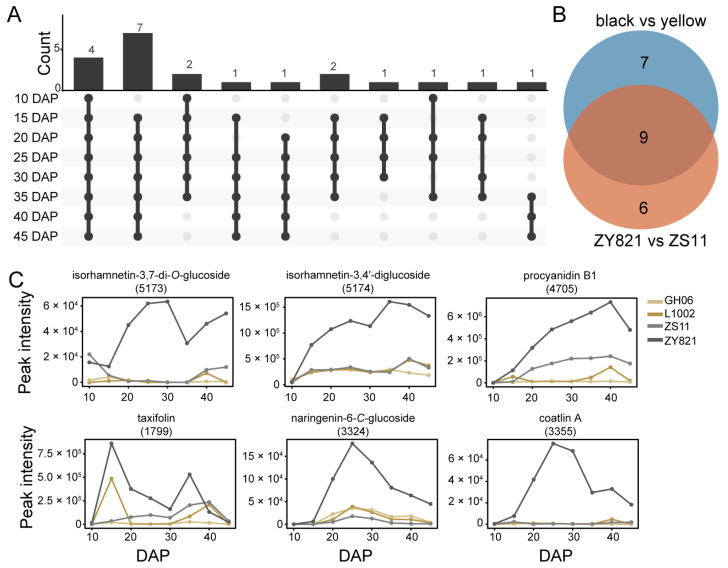
Differences in flavonoid features between ZY821 and ZS11. (**A**) Upset plots showing the differentially abundant flavonoid features between ZY821 and ZS11 across seed development. (**B**) Classification of upregulated and downregulated features common from 20 days after pollination (DAP) to 45 DAP. (**C**) Accumulation profiles of six differentially abundant features in the four *B. napus* varieties.

To better understand the differences in flavonoid contents between the two black-seeded *B. napus* varieties, we identified the differentially abundant flavonoid features between these two varieties, using VIP > 1 and log2(FC) > 1 as selection criteria. There were 15 flavonoid features with a higher accumulation in ZY821 than in ZS11 during at least six seed developmental stages (Figure 4A). Nine of these features belonged to the previously identified differentially abundant features between yellow-seeded and black-seeded *B. napus* varieties (Figure 4B). These features were mainly epicatechin and procyanidin compounds (Figure 3E). The remaining six features accumulated to high levels in ZY821 seeds, especially coatlin A (feature 3355) and isorhamnetin-3,7-di-*O*-glucoside (features 5173), which were present at very low levels in ZS11 seeds (Figure 4C, Supplementary Data S4). These results indicate that ZS11 seeds accumulate lower levels of flavonoids than ZY821 seeds, especially for compounds derived from flavonoid compounds, which may be the reason why the oil content of ZY821 is lower than that of ZS11.

### 2.5. Combining Metabolome and Multiple Transcriptome Datasets to Identify Seed Pigmentation Regulatory Genes

**Figure 5:**
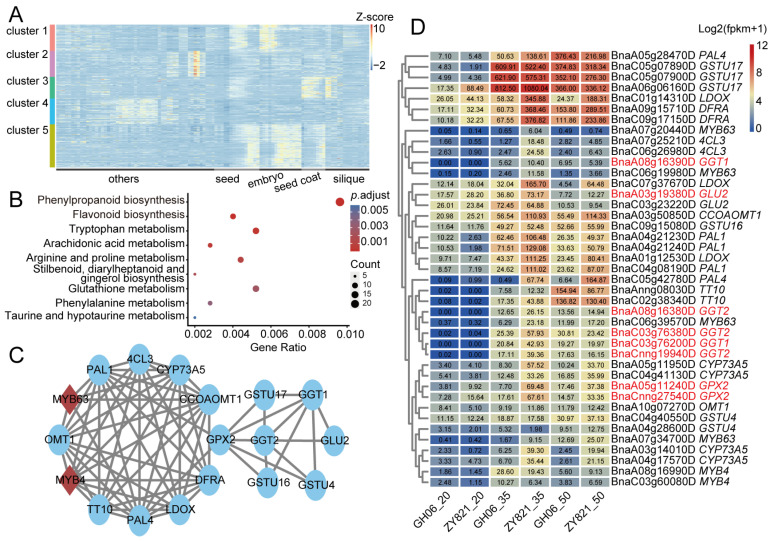
Gene regulatory gene network of flavonoid features. (**A**) Heatmap representation and clustering analysis of candidate genes’ expression levels in different tissues of ZS11 variety. Data downloaded from BrassicaEDB. (**B**) KEGG pathway enrichment analysis of genes from cluster 3 that are specifically highly expressed in the seed coat. (**C**) Regulatory network of the flavonoid core genes in cluster 3. Red diamonds indicate transcription factors. (**D**) Heatmap representation of expression levels for genes from cluster 3 in different yellow-seeded or black-seeded *B. napus* varieties. Red texts indicate differentially expressed glutathione (GSH)-related genes between *B. napus* varieties with yellow or black seeds.

To identify genes that may regulate the biosynthesis and/or accumulation of the 16 differentially abundant flavonoid features between yellow-seeded and black-seeded *B. napus* varieties, we integrated transcriptome and metabolome data produced across ZS11 seed development and identified 7223 candidate genes. A k-means clustering analysis of these candidate genes showed that the genes in cluster 3 are specifically expressed in the seed coat and in seeds (Figure 5A). This finding suggests that the genes in cluster 3 are likely to be related to the accumulation of seed coat pigments. A Kyoto Encyclopedia of Genes and Genomes (KEGG) pathway enrichment analysis revealed that the genes from cluster 3 are enriched in the ‘phenylpropanoid biosynthesis’ and ‘flavonoid biosynthesis’ pathways (Figure 5B). We also submitted the genes from cluster 3 to the STRING database to construct a protein–protein interaction (PPI) network, which helped us draw a gene regulatory network that may regulate seed color, including genes homologous to *Arabidopsis MYB4*, *MYB63*, *4CL3*, *O-METHYLTRANSFERASE 1* (*OMT1*), *CAFFEOYL COENZYME A O-METHYLTRANSFERASE 1* (*CCoAOMT1*), *CYTOCHROME P450 73A5* (*CYP73A5*), *PHENYLALANINE AMMONIA-LYASE 1* (*PAL1*), *PAL4*, *DFRA*, *LEUCOANTHOCYANIDIN DIOXYGENASE* (*LDOX*), and *TT10* (Figure 5C). In addition, seven glutathione (GSH)-related genes were included, being homologous to *Arabidopsis GLUTATHIONE PEROXIDASE 2* (*GPX2*), *GLUTAMATE SYNTHASE 2* (*GLU2*), *GAMMA-GLUTAMYL TRANSPEPTIDASE 1* (*GGT1*), *GGT2*, *GLUTATHIONE S-TRANSFERASE TAU 4* (*GSTU4*), *GSTU16*, and *GSTU17* (Figure 5C). To further determine the relationship between these genes and seed coat color, we extracted the expression levels of these genes in yellow-seeded and black-seeded *B. napus* varieties from previously published data [15]. The genes homologous to flavonoid pathway genes showed differential expression between yellow-seeded and black-seeded *B. napus* varieties, as did those homologous to *GLU2*, *GPX2*, *GGT1*, and *GGT2* (Figure 5D). These results suggest that glutathione (GSH)-related genes were co-expressed with flavonoid pathway genes, and glutathione may play an important role in the transport of flavonoids.

### 2.6. Construction of Molecular Networks to Explore Unknown Features

**Figure 6:**
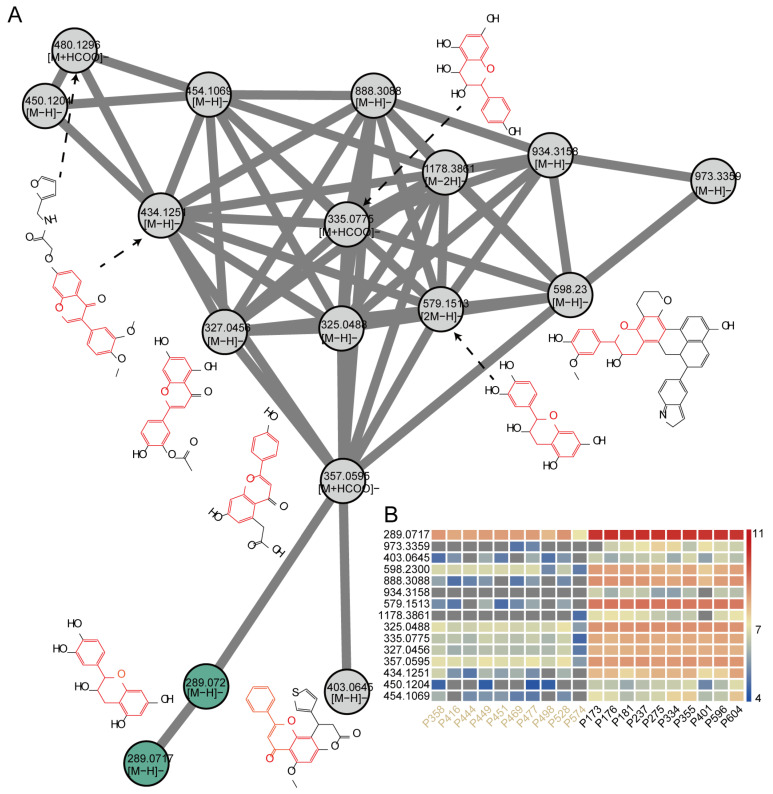
Molecular Network 1. (**A**) Molecular network 1, extracted from 130 upregulated features. Green nodes represent known metabolites, and gray nodes represent unknown metabolites. The red color in the chemical structure represents the C6-C3-C6 rings. (**B**) Heatmap representation of the accumulation of features in molecular network 1 in 10 yellow-seeded and 10 black-seeded *B. napus* varieties. Feature intensities were converted using log10. The names in the columns were assigned based on the *m*/*z* values of features in (**A**).

Only 21 metabolites were annotated among the 130 features upregulated in black-seeded *B. napus* varieties compared to yellow-seeded *B. napus* varieties. To enrich our knowledge of unknown differentially abundant features, we analyzed these 130 features using molecular networks, producing two molecular networks containing flavonoid features, named molecular network 1 (MN1) and MN2. MN1 consisted of 17 features, including two epicatechin features. We predicted the chemical structures of the 15 other features by MS-finder. Importantly, seven of the 15 features were predicted to contain the C6-C3-C6 rings typical of flavonoid compounds (Figure 6A). To verify the reliability of these unidentified features in relation to seed color, we determined their relative contents using previously published metabolic data from the seeds of yellow-seeded and black-seeded *B. napus* varieties [15]. We successfully detected 14 out of these 15 features in the datasets; notably, these features all accumulated to higher levels in black-seeded *B. napus* varieties than in yellow-seeded *B. napus* varieties (Figure 6B). We used the same pipeline to analyze MN2, which contained six proanthocyanidin features (Figure S3A). Eight of the 29 features in MN2 were predicted to contain the C6-C3-C6 rings of flavonoid compounds (Figure S3A). The accumulation of the 29 features in MN2 was also higher in black-seeded *B. napus* varieties than in yellow-seeded *B. napus* varieties (Figure S3B). These results indicate that the 38 unknown features identified by the molecular networks contribute to the pigmentation of the *B. napus* seed coat.

## 3. Discussion

Seeds are the major tissue harvested from *B. napus* varieties. The composition of compounds in their seeds has always attracted much attention: this is especially true for phenolic acids, which are involved in grain color formation and have antioxidant activity [33,34]. To obtain a more detailed metabolic profile of *B. napus* seeds, we performed a metabolomic analysis of seeds from four *B. napus* varieties at eight developmental stages. We detected a total of 4,540 features, of which 366 were annotated as known metabolites (Supplementary Data S2). Overall, our metabolic data show the characteristics of seed development stage and seed color in *B. napus* (Figure 2B). In particular, samples seeds collected from 10 DAP to 25 DAP have showed greater metabolic differences than seeds collected from 30 DAP to 45 DAP. (Figure 2C). This result may reflect the fact that *B. napus* seeds have largely completed their development after 30 DAP, after which time nutrients such as oil begin to accumulate, resulting in a decrease in the overall metabolic activity of seeds.

Studies on crop species with yellow seeds such as *B. rapa* [20,35], *B. juncea* [36,37], and *B. napus* [22] have shown that the blockage of the procyanidin biosynthesis pathway is the main reason for their seed color. Our results show that in the early stage of seed development (10 DAP to 20 DAP), epicatechin and procyanidin contents showed differences in their accumulation pattern between yellow-seeded and black-seeded *B. napus* varieties, differences that remain until seed development is completed (Figure 3E). Therefore, 10–20 DAP may be a critical period for the accumulation of flavonoids in the *B. napus* seed coat. In addition, we identified some metabolites upstream of the flavonoid pathway that highly accumulated in black-seeded *B. napus* varieties, such as phloretin (feature 1492). This type of flavonoid may subsequently participate in the establishment of seed coat color in *B. napus* by oxidation into melanin [28]. It is worth noting that, similar to the accumulation pattern of soluble anthocyanins in *B. napus* seeds determined by Auger [33], the accumulation of these differentially abundant metabolites decreased in the late stage of seed development (35–45 DAP) in black-seeded varieties. We speculate that these metabolites may become oxidized and consumed by laccase and phenol oxidase in the late stage of seed development to form the seed coat color [38,39].

Meanwhile, we observed the distinct accumulation patterns of flavonoid glucosides. Isorhamnetin-3-*O*-glucoside-sulfate (feature 4548) accumulated more in black-seeded rapeseed, whereas the cyanidin-3,5-*O*-glucoside (feature 6054) derivative accumulated more in yellow-seeded rapeseed (Figure 3E). Additionally, isorhamnetin-3,7-di-*O*-glucoside (feature 5173), isorhamnetin-3,4′-di-glucoside (feature 5174), and naringenin-6-*C*-glucoside (feature 3324) accumulated more in ZY821 than in the other three varieties (Figure 4C). Flavonoid glucosides perform multiple functions, including defense against pathogens [40], UV protection [10,41], and cold protection [42,43], and are typically catalyzed by the UGT gene family [44]. However, no UGT genes were identified during the candidate gene screening process. Investigating whether flavonoid glucosides in rapeseed contribute to seed coat pigment formation and whether the UGT gene family plays a role in this process could offer valuable insights for enhancing rapeseed seed quality. In addition, we predicted 38 possible flavonoid features, which showed stable accumulation differences between yellow-seeded and black-seeded *B. napus* varieties (Figure 6B and Figure S3B). Therefore, in addition to proanthocyanidins, many flavonoid compounds are involved in the formation of seed coat color in *B. napus*.

The content of flavonoid compounds in *B. napus* seeds is negatively correlated with oil content [45]. The oil content of *B. napus* seeds can be increased by knocking out genes in the flavonoid biosynthesis pathway, while resulting in a different seed coat color pattern depending on the gene being knocked out, owing to the complexity of the flavonoid biosynthesis pathway [19,23,24]. In this study, the flavonoid pathways of the two black-seeded *B. napus* varieties showed large differences. The accumulation of flavonoid metabolites in the high-oil variety ZS11 was much lower than that in the low-oil material ZY821, especially isorhamnetin-3,7-di-*O*-glucoside (features 5173), isorhamnetin-3,4′-diglucoside (features 5174), naringenin-6-*C*-glucoside (feature 3324), and coatlin A (feature 3355), which accumulated very little in ZS11 seeds (Figure 3E and Figure 4C). The low accumulation of these flavonoid metabolites may be one of the reasons why the oil content of ZS11 is higher than that of ZY821. The study of Li et al. reached a similar conclusion; that is, knocking out the seed color candidate gene *BnaC05.UK* decreased the content of naringenin and increased the oil content of *B. napus* seeds, while seed color remained black [45]. These results show that inhibiting the flavonoid pathway does not necessarily lead to yellow-seeded *B. napus* varieties, but can increase the oil content. Improving the quality of *B. napus* seeds by regulating the metabolic level of the flavonoid pathway may be an important direction for subsequent *B. napus* breeding.

The flavonoid pathway of *B. napus* is somewhat conserved with that of Arabidopsis, as most of the *B. napus* homologs to the genes in the flavonoid pathway of Arabidopsis modulate the accumulation of flavonoids in *B. napus* [19,23,24,45]. At present, the catalytic function of the core genes of the flavonoid pathway has long been clear. In this study, we identified 12 flavonoid catalytic genes (Figure 5C). These genes are not only specifically expressed in seeds and seed coats, but also exhibited vast expression differences between yellow-seeded and black-seeded *B. napus* varieties (Figure 5A,D). In particular, these genes contain *PAL* and *4CL*, which are key genes in the phenylpropanoid pathway upstream of the flavonoid pathway [12,14]. This suggests that the flavonoid pathway in yellow-seeded rapeseed is inhibited, not at a single metabolic step, but throughout the entire metabolic pathway. In addition, we identified three glutathione-related genes that followed similar expression trends as the above flavonoid-related genes (Figure 5C,D). On the one hand, GSH is considered to stimulate the accumulation of flavonoids in plant cells [46,47]. On the other hand, GSH is an important molecule that the anthocyanin transporter ABCC1 depends on [48]. GSH may therefore play an important role in the formation of *B. napus* seed coat pigmentation, but its specific mechanism remains to be experimentally assessed.

## 4. Materials and Methods

### 4.1. Plant Materials

The four *B. napus* varieties used in this study, ‘ZS11’, ‘ZY821’, ‘GH06’, and ‘L1002’, were sown in late September 2021 and grown in the experimental field in Beibei (N29°76′, E106°38′; Chongqing, China) under normal field conditions. Immature seeds were harvested from five individual plants per variety at 10, 15, 20, 25, 30, 35, 40, and 45 days after pollination (DAP). The seeds were divided into three portions, collected into 2 mL centrifuge tubes, immediately frozen in liquid nitrogen, and then stored at −80 °C until metabolite extraction.

### 4.2. Metabolite Extraction

The extraction of metabolites from developing seeds was performed as previously described [22], with slight modifications. Fresh seed powder (100 mg) was weighed into a microfuge tube pre-cooled in liquid nitrogen (2 mL; Eppendorf, Leipzig, Germany), and homogenized in aqueous methanol (1 mL; 80% [*v*/*v*] methanol in water). Samples were then sonicated (KQ-100E, Kunshan Ultrasonic Instrument Co., Ltd., Kunshan, China) for 1 h, and the crude extracts were centrifuged for 15 min with 10,000× *g* at 4 °C. Finally, the supernatants were filtered through a 0.22-µm nylon filter and analyzed via ultrahigh-performance liquid chromatography–heated electrospray ionization–tandem mass spectrometry (UPLC–HESI–MS/MS). All samples were analyzed in three biological replicates.

### 4.3. UHPLC−HESI−MS/MS Analysis

The chromatography conditions were consistent with those described by Qu et al. [22]. After filtering, 10 μL of each sample was analyzed using a Dionex UltiMateTM 3000 UHPLC system (Thermo Fisher Scientific, Waltham, MA, USA) connected to a Thermo Scientific Q-Exactive System equipped with an S-Lens ionizer source (Thermo Scientific, USA) in negative mode. An Acquity UPLC BEH C18 chromatography column (150 mm × 2.1 mm, 1.7 μm particle size) (Waters, Wexford, Ireland) was used with a guard column (Acquity UPLC BEH C18 1.7 μm VanGuard Pre-Column 5 mm × 2.1 mm; Waters) controlled at 30 °C. The composition of the mobile phase was as follows: solvent A (0.1% [*v*/*v*] formic acid in water) and solvent B (0.1% [*v*/*v*] formic acid in acetonitrile). The following mobile phase gradient was employed: 0–2 min, 5–10% solvent B; 2–10 min, 10–25% solvent B; 10–15 min, 25–50% solvent B; 15–20 min, 50–95% solvent B; 20–23 min, 95% solvent B; 23–23.5 min, 95–5% solvent B; and 23.5–28 min, 5% solvent B. The flow rate was set to 0.300 mL·min^−1^.

The spectra were recorded in full scan mode, covering a mass range from *m*/*z* 100 to 1200. The operation parameters were as follows: source voltage, 3.5 kV; sheath gas, 35 (arbitrary units); auxiliary gas, 10 (arbitrary units); sweep gas, 0 (arbitrary units); and capillary temperature, 350 °C.

### 4.4. Metabolomics Data Processing

All raw data were analyzed using MS-DIAL v4.60 software with three mass databases MoNA, MSMS_Public_EXP_VS17, and MSMS_Public_ExpBioInsilico_VS17 accessed on 2 November 2022. (https://systemsomicslab.github.io/compms/msdial/main.html#MSP) [49]. The parameters of MS-DIAL were modified based on the previously published studies [50], MS1 tolerance: 0.002 Da, MS2 tolerance: 0.002 Da, and minimum peak height: 500,000. Metabolite identification was: MS1 tolerance: 0.002 Da, MS2 tolerance: 0.002 Da, the minimum number of matched peaks: 0.5. The SMILES (Simplified Molecular Input Line Entry Specification) IDs of the identified features were used to identify the class of compounds using NPClassfire [51] via chemodiv [52]. The feature molecular network was established with the Global Natural Products Social Molecular Networking (GNPS) platform with the mgf file as input [53], and visualization with Cytoscape v3.9 [54]. MS-finder was used to predict the chemical structure of unknown compounds [55].

### 4.5. Correlation Analysis of Metabolome and Transcriptome Data

Transcriptome data for ZS11 were downloaded from BrassicaEDB [56] accessed on 15 January 2023. The R package dtw was used to calculate the similarity of features to gene trends [57]. In the results for each feature, the top 2000 genes as determined by their Warping distance calculated by dtw were considered to be associated with that feature [58]. The genes that were associated with at least two differentially abundant (DA) features were selected as candidate genes.

### 4.6. Identification of Core Genes

The candidate genes identified above were analyzed using the R package fpc to identify gene clusters (https://cran.r-project.org/package=fpc, accessed on 17 January 2023). The gene IDs from each cluster were uploaded to the BnIR website for Kyoto Encyclopedia of Genes and Genomes (KEGG) pathway enrichment analysis [59]. The protein sequences corresponding to the genes in each cluster are uploaded to the STRING database website and mapped against Arabidopsis thaliana for a protein–protein interaction (PPI) network analysis (https://string-db.org/, accessed on 25 January 2023). The PPI network was imported to Cytoscape [54], and highly interconnected regions within the network were identified using the Molecular Complex Detection (MCODE) v2.0.3 app [60].

### 4.7. Statistical Analysis

The principal component analysis (PCA) and the orthogonal partial least-squares discriminant analysis (OPLS-DA) were performed by metaboanalyst 5.0 software [61]. The features with a variable importance in projection value (VIP) > 1 and with a fold-change (FC) above 2 (upregulated) or below 0.5 (downregulated) were considered to be differentially abundant features. When comparing yellow-seeded and black-seeded rapeseed, yellow-seeded rapeseed was used as the control, while ZS11 was used as the control when comparing ZS11 and ZY821.

## 5. Conclusions

In conclusion, we conclude that the complex accumulation patterns of other flavonoid derivatives in yellow-seeded and black-seeded *B. napus* varieties is the reason for the complexity of *B. napus* seed coat color, and that these metabolites affect the oil content from the perspective of carbon source distribution. Finally, the role of glutathione in *B. napus* seed coat color deserves further study. This study lays the foundation for improving *B. napus* seed and oil quality by regulating the flavonoid metabolic pathway in the future.

## Data Availability

All additional datasets supporting the findings of this study are included within the article and Supplementary Materials.

## Supplementary Materials

The following supporting information can be downloaded at: https://www.mdpi.com/article/10.3390/plants14050753/s1, Figure S1. Correlation matrix of all quality control samples. Figure S2. UpSet showing relationships of up (A) and down (B) abundant features between *B. napus* varieties with yellow or black seeds across seed development. Figure S3. Molecular Network 2. (A) Molecular network 2, extracted from 130 upregulated features. Green nodes represent known metabolites, and gray nodes represent unknown metabolites. (B) Heatmap representation of the accumulation of features in molecular network 2 in 10 yellow-seeded and 10 black-seeded *B. napus* varieties. Feature intensities were converted using log10. The names in the columns were assigned based on the m/z values of features in (A). Data S1. Near-infrared scanning results for 15 quality traits of four *B. napus* varieties. Data S2. Information of all detected features. Data S3. Intensities of differentially abundant flavonoid features between yellow-seeded and black-seeded *Brassica napus* across four varieties. Data S4. Intensities of differentially abundant flavonoid features between ZY821 and ZS11 across four *Brassica napus* varieties.

## References

[1] Maeda H.A., Fernie A.R. (2021). Evolutionary History of Plant Metabolism. Annu. Rev. Plant Biol..

[2] Erb M., Kliebenstein D.J. (2020). Plant Secondary Metabolites as Defenses, Regulators, and Primary Metabolites: The Blurred Functional Trichotomy. Plant Physiol..

[3] Fang C.Y., Fernie A.R., Luo J. (2019). Exploring the Diversity of Plant Metabolism. Trends Plant Sci..

[4] Wang S.C., Alseekh S., Fernie A.R., Luo J. (2019). The Structure and Function of Major Plant Metabolite Modifications. Mol. Plant.

[5] Zhang S., Wu Y., Ren Y., Xu Y., An H., Zhao Q., Wang Y., Li H. (2024). Widely metabolomic combined with transcriptome analysis to build a bioactive compound regulatory network for the fruit growth cycle in *Pseudocydonia sinensis*. Food Chem..

[6] Zhou L., Wu S., Chen Y., Huang R., Cheng B., Mao Q., Liu T., Liu Y., Zhao K., Pan H. (2024). Multi-omics analyzes of *Rosa gigantea* illuminate tea scent biosynthesis and release mechanisms. Nat. Commun..

[7] Cao Y., Han Z., Zhang Z., He L., Huang C., Chen J., Dai F., Xuan L., Yan S., Si Z. (2024). UDP-glucosyltransferase 71C4 controls the flux of phenylpropanoid metabolism to shape cotton seed development. Plant Commun..

[8] Dan Z., Chen Y., Li H., Zeng Y., Xu W., Zhao W., He R., Huang W. (2021). The metabolomic landscape of rice heterosis highlights pathway biomarkers for predicting complex phenotypes. Plant Physiol..

[9] Chen S., Sun B., Shi Z.Y., Miao X.X., Li H.C. (2022). Identification of the rice genes and metabolites involved in dual resistance against brown planthopper and rice blast fungus. Plant Cell Environ..

[10] Peng M., Shahzad R., Gul A., Subthain H., Shen S., Lei L., Zheng Z., Zhou J., Lu D., Wang S. (2017). Differentially evolved glucosyltransferases determine natural variation of rice flavone accumulation and UV-tolerance. Nat. Commun..

[11] Alseekh S., Scossa F., Wen W., Luo J., Yan J., Beleggia R., Klee H.J., Huang S., Papa R., Fernie A.R. (2021). Domestication of Crop Metabolomes: Desired and Unintended Consequences. Trends Plant Sci..

[12] Tohge T., de Souza L.P., Fernie A.R. (2017). Current understanding of the pathways of flavonoid biosynthesis in model and crop plants. J. Exp. Bot..

[13] Shen N., Wang T., Gan Q., Liu S., Wang L., Jin B. (2022). Plant flavonoids: Classification, distribution, biosynthesis, and antioxidant activity. Food Chem..

[14] Liu W., Feng Y., Yu S., Fan Z., Li X., Li J., Yin H. (2021). The Flavonoid Biosynthesis Network in Plants. Int. J. Mol. Sci..

[15] Zhao J., Dixon R.A. (2010). The ’ins’ and ’outs’ of flavonoid transport. Trends Plant Sci..

[16] Lepiniec L., Debeaujon I., Routaboul J.M., Baudry A., Pourcel L., Nesi N., Caboche M. (2006). Genetics and biochemistry of seed flavonoids. Annu. Rev. Plant Biol..

[17] Chen Y.Y., Lu H.Q., Jiang K.X., Wang Y.R., Wang Y.P., Jiang J.J. (2022). The Flavonoid Biosynthesis and Regulation in *Brassica napus*: A Review. Int. J. Mol. Sci..

[18] Petraru A., Amariei S. (2024). Rapeseed—An Important Oleaginous Plant in the Oil Industry and the Resulting Meal a Valuable Source of Bioactive Compounds. Plants.

[19] Li H., Yu K., Zhang Z., Yu Y., Wan J., He H., Fan C. (2024). Targeted mutagenesis of flavonoid biosynthesis pathway genes reveals functional divergence in seed coat colour, oil content and fatty acid composition in *Brassica napus* L.. Plant Biotechnol. J..

[20] Zhao H., Shang G., Yin N., Chen S., Shen S., Jiang H., Tang Y., Sun F., Zhao Y., Niu Y. (2022). Multi-omics analysis reveals the mechanism of seed coat color formation in *Brassica rapa* L.. Theor. Appl. Genet..

[21] Yu C.Y. (2013). Molecular mechanism of manipulating seed coat coloration in oilseed *Brassica* species. J. Appl. Genet..

[22] Qu C., Yin N., Chen S., Wang S., Chen X., Zhao H., Shen S., Fu F., Zhou B., Xu X. (2020). Comparative Analysis of the Metabolic Profiles of Yellow- versus Black-Seeded Rapeseed Using UPLC-HESI-MS/MS and Transcriptome Analysis. J. Agric. Food Chem..

[23] Zhai Y., Yu K., Cai S., Hu L., Amoo O., Xu L., Yang Y., Ma B., Jiao Y., Zhang C. (2020). Targeted mutagenesis of *BnTT8* homologs controls yellow seed coat development for effective oil production in *Brassica napus* L.. Plant Biotechnol. J..

[24] Xie T., Chen X., Guo T., Rong H., Chen Z., Sun Q., Batley J., Jiang J., Wang Y. (2020). Targeted Knockout of *BnTT2* Homologues for Yellow-Seeded *Brassica napus* with Reduced Flavonoids and Improved Fatty Acid Composition. J. Agric. Food Chem..

[25] Lim G.H., Singhal R., Kachroo A., Kachroo P. (2017). Fatty Acid- and Lipid-Mediated Signaling in Plant Defense. Annu. Rev. Phytopathol..

[26] Marles M.S., Gruber M.Y. (2004). Histochemical characterisation of unextractable seed coat pigments and quantification of extractable lignin in the Brassicaceae. J. Sci. Food Agric..

[27] Yin N., Li B., Liu X., Liang Y., Lian J., Xue Y., Qu C., Lu K., Wei L., Wang R. (2022). Two types of cinnamoyl-CoA reductase function divergently in accumulation of lignins, flavonoids and glucosinolates and enhance lodging resistance in *Brassica napus*. Crop J..

[28] Li B., Jia Y., Xu L., Zhang S., Long Z., Wang R., Guo Y., Zhang W., Jiao C., Li C. (2024). Transcriptional convergence after repeated duplication of an amino acid transporter gene leads to the independent emergence of the black husk/pericarp trait in barley and rice. Plant Biotechnol. J..

[29] Yang C., Shen S., Zhou S., Li Y., Mao Y., Zhou J., Shi Y., An L., Zhou Q., Peng W. (2022). Rice metabolic regulatory network spanning the entire life cycle. Mol. Plant.

[30] Li Y., Chen Y., Zhou L., You S., Deng H., Chen Y., Alseekh S., Yuan Y., Fu R., Zhang Z. (2020). MicroTom Metabolic Network: Rewiring Tomato Metabolic Regulatory Network throughout the Growth Cycle. Mol. Plant.

[31] Shu P., Zhang Z., Wu Y., Chen Y., Li K., Deng H., Zhang J., Zhang X., Wang J., Liu Z. (2023). A comprehensive metabolic map reveals major quality regulations in red-flesh kiwifruit (*Actinidia chinensis*). New Phytol..

[32] Zeng Z., Li Y., Zhu M., Wang X., Wang Y., Li A., Chen X., Han Q., Nieuwenhuizen N.J., Ampomah-Dwamena C. (2025). Kiwifruit spatiotemporal multiomics networks uncover key tissue-specific regulatory processes throughout the life cycle. Plant Physiol..

[33] Auger B., Marnet N., Gautier V., Maia-Grondard A., Leprince F., Renard M., Guyot S., Nesi N., Routaboul J.-M. (2010). A Detailed Survey of Seed Coat Flavonoids in Developing Seeds of *Brassica napus* L.. J. Agric. Food Chem..

[34] Shao Y., Jiang J., Ran L., Lu C., Wei C., Wang Y. (2014). Analysis of flavonoids and hydroxycinnamic acid derivatives in rapeseeds (*Brassica napus* L. var. napus) by HPLC-PDA--ESI(--)-MS(n)/HRMS. J. Agric. Food Chem..

[35] Ren Y., Zhang N., Li R., Ma X., Zhang L. (2021). Comparative transcriptome and flavonoids components analysis reveal the structural genes responsible for the yellow seed coat color of *Brassica rapa* L.. PeerJ.

[36] Shen S., Tang Y., Zhang C., Yin N., Mao Y., Sun F., Chen S., Hu R., Liu X., Shang G. (2021). Metabolite Profiling and Transcriptome Analysis Provide Insight into Seed Coat Color in *Brassica juncea*. Int. J. Mol. Sci..

[37] Qian L., Yang L., Liu X., Wang T., Kang L., Chen H., Lu Y., Zhang Y., Yang S., You L. (2025). Natural variations in *TT8* and its neighboring *STK* confer yellow seed with elevated oil content in *Brassica juncea*. Proc. Natl. Acad. Sci. USA.

[38] Pourcel L., Routaboul J.M., Kerhoas L., Caboche M., Lepiniec L., Debeaujon I. (2005). *TRANSPARENT TESTA10* encodes a laccase-like enzyme involved in oxidative polymerization of flavonoids in *Arabidopsis* seed coat. Plant Cell.

[39] Janusz G., Pawlik A., Świderska-Burek U., Polak J., Sulej J., Jarosz-Wilkołazka A., Paszczyński A. (2020). Laccase Properties, Physiological Functions, and Evolution. Int. J. Mol. Sci..

[40] Yuan Z., Li G., Zhang H., Peng Z., Ding W., Wen H., Zhou H., Zeng J., Chen J., Xu J. (2024). Four novel *Cit7GlcTs* functional in flavonoid 7-O-glucoside biosynthesis are vital to flavonoid biosynthesis shunting in citrus. Hortic. Res..

[41] Zhang F., Guo H., Huang J., Yang C., Li Y., Wang X., Qu L., Liu X., Luo J. (2020). A UV-B-responsive glycosyltransferase, *OsUGT706C2*, modulates flavonoid metabolism in rice. Sci. China: Life Sci..

[42] Bao H., Yuan L., Luo Y., Jing X., Zhang Z., Wang J., Zhu G. (2024). A freezing responsive UDP-glycosyltransferase improves potato freezing tolerance via modifying flavonoid metabolism. Hortic. Plant J..

[43] Liu X., Wang T., Ruan Y., Xie X., Tan C., Guo Y., Li B., Qu L., Deng L., Li M. (2024). Comparative Metabolome and Transcriptome Analysis of Rapeseed (*Brassica napus* L.) Cotyledons in Response to Cold Stress. Plants.

[44] Wilson A.E., Tian L. (2019). Phylogenomic analysis of UDP-dependent glycosyltransferases provides insights into the evolutionary landscape of glycosylation in plant metabolism. Plant J..

[45] Li L., Tian Z., Chen J., Tan Z., Zhang Y., Zhao H., Wu X., Yao X., Wen W., Chen W. (2023). Characterization of novel loci controlling seed oil content in *Brassica napus* by marker metabolite-based multi-omics analysis. Genome Biol..

[46] Au K.K., Pérez-Gómez J., Neto H., Müller C., Meyer A.J., Fricker M.D., Moore I. (2012). A perturbation in glutathione biosynthesis disrupts endoplasmic reticulum morphology and secretory membrane traffic in *Arabidopsis thaliana*. Plant J..

[47] Shelton D., Stranne M., Mikkelsen L., Pakseresht N., Welham T., Hiraka H., Tabata S., Sato S., Paquette S., Wang T.L. (2012). Transcription factors of Lotus: Regulation of isoflavonoid biosynthesis requires coordinated changes in transcription factor activity. Plant Physiol..

[48] Francisco R.M., Regalado A., Ageorges A., Burla B.J., Bassin B., Eisenach C., Zarrouk O., Vialet S., Marlin T., Chaves M.M. (2013). ABCC1, an ATP binding cassette protein from grape berry, transports anthocyanidin 3-O-Glucosides. Plant Cell.

[49] Tsugawa H., Ikeda K., Takahashi M., Satoh A., Mori Y., Uchino H., Okahashi N., Yamada Y., Tada I., Bonini P. (2020). A lipidome atlas in MS-DIAL 4. Nat. Biotechnol..

[50] Beck L.C., Masi A.C., Young G.R., Vatanen T., Lamb C.A., Smith R., Coxhead J., Butler A., Marsland B.J., Embleton N.D. (2022). Strain-specific impacts of probiotics are a significant driver of gut microbiome development in very preterm infants. Nat. Microbiol..

[51] Kim H.W., Wang M., Leber C.A., Nothias L.F., Reher R., Kang K.B., van der Hooft J.J.J., Dorrestein P.C., Gerwick W.H., Cottrell G.W. (2021). NPClassifier: A Deep Neural Network-Based Structural Classification Tool for Natural Products. J. Nat. Prod..

[52] Petrén H., Köllner T.G., Junker R.R. (2023). Quantifying chemodiversity considering biochemical and structural properties of compounds with the R package chemodiv. New Phytol..

[53] Wang M., Carver J.J., Phelan V.V., Sanchez L.M., Garg N., Peng Y., Nguyen D.D., Watrous J., Kapono C.A., Luzzatto-Knaan T. (2016). Sharing and community curation of mass spectrometry data with Global Natural Products Social Molecular Networking. Nat. Biotechnol..

[54] Shannon P., Markiel A., Ozier O., Baliga N.S., Wang J.T., Ramage D., Amin N., Schwikowski B., Ideker T. (2003). Cytoscape: A software environment for integrated models of biomolecular interaction networks. Genome Res..

[55] Lai Z., Tsugawa H., Wohlgemuth G., Mehta S., Mueller M., Zheng Y., Ogiwara A., Meissen J., Showalter M., Takeuchi K. (2018). Identifying metabolites by integrating metabolome databases with mass spectrometry cheminformatics. Nat. Methods.

[56] Chao H., Li T., Luo C., Huang H., Ruan Y., Li X., Niu Y., Fan Y., Sun W., Zhang K. (2020). BrassicaEDB: A Gene Expression Database for Brassica Crops. Int. J. Mol. Sci..

[57] Giorgino T. (2009). Computing and Visualizing Dynamic Time Warping Alignments in R: The dtw Package. J. Stat. Softw..

[58] Conway J.R., Lex A., Gehlenborg N. (2017). UpSetR: An R package for the visualization of intersecting sets and their properties. Bioinformatics.

[59] Yang Z., Wang S., Wei L., Huang Y., Liu D., Jia Y., Luo C., Lin Y., Liang C., Hu Y. (2023). BnIR: A multi-omics database with various tools for *Brassica napus* research and breeding. Mol. Plant.

[60] Bader G.D., Hogue C.W. (2003). An automated method for finding molecular complexes in large protein interaction networks. BMC Bioinform..

[61] Pang Z., Zhou G., Ewald J., Chang L., Hacariz O., Basu N., Xia J. (2022). Using MetaboAnalyst 5.0 for LC-HRMS spectra processing, multi-omics integration and covariate adjustment of global metabolomics data. Nat. Protoc..

