# Supplementary figures and images for: Untargeted Metabolomics Analysis Reveals Differential Accumulation of Flavonoids Between Yellow-Seeded and Black-Seeded Rapeseed Varieties
Source: Plants (Basel). 2025 Mar 1;14(5):753. doi: 10.3390/plants14050753 (PMC11902209; doi:10.3390/plants14050753)

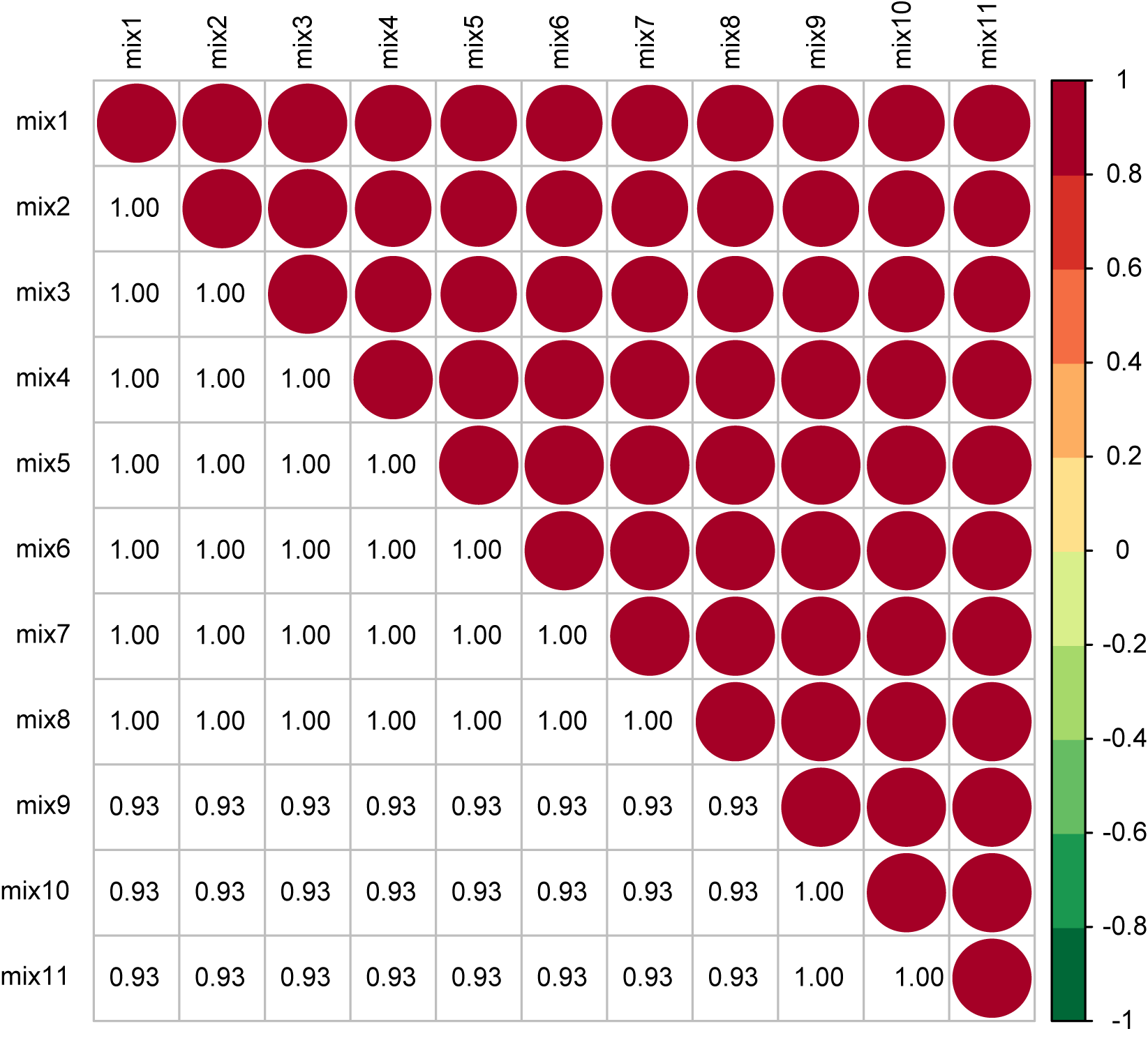

Supplement: Supplementary file 1 [file plants-14-00753-s001.zip › FigS1.tif]

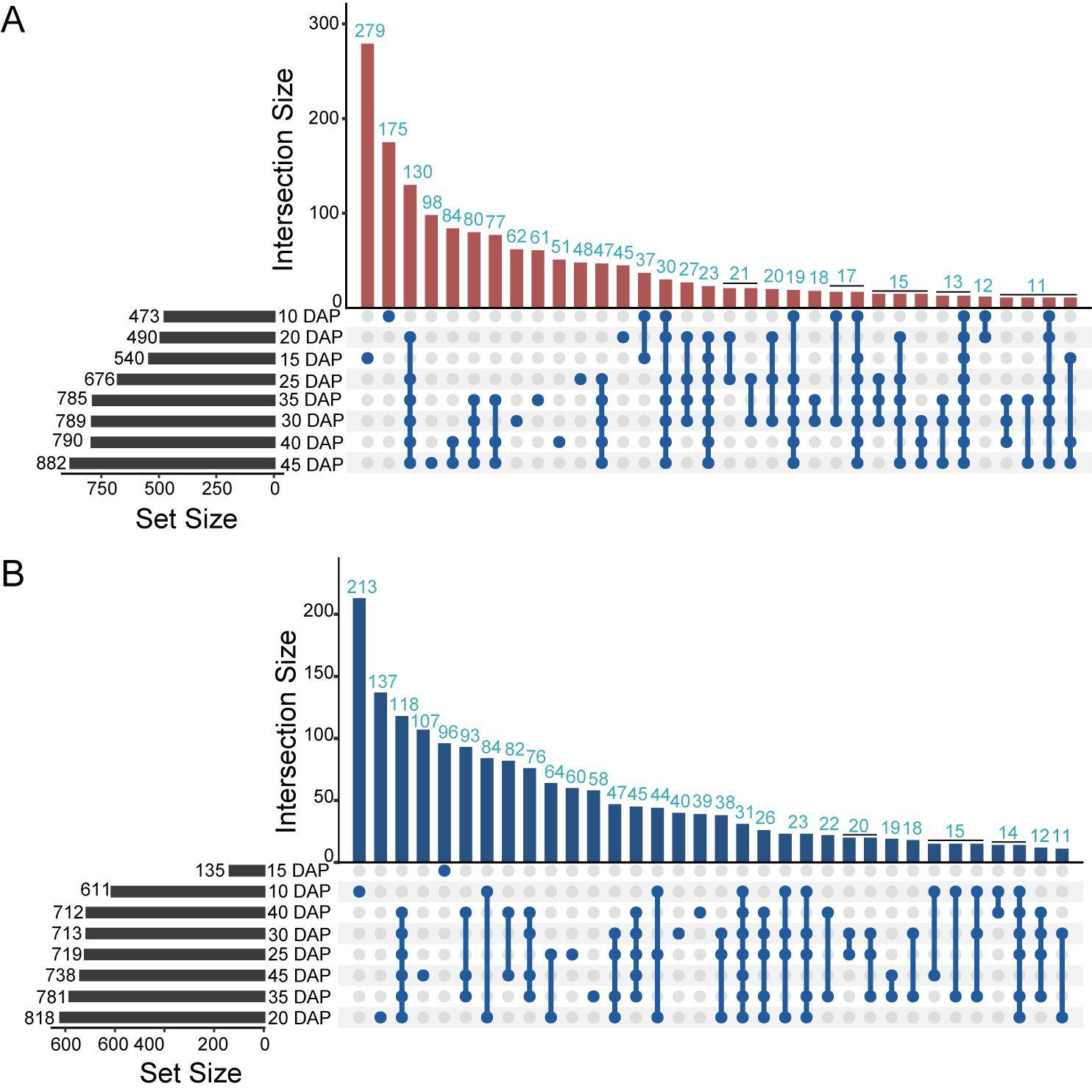

Supplement: Supplementary file 1 [file plants-14-00753-s001.zip › FigS2.tif]

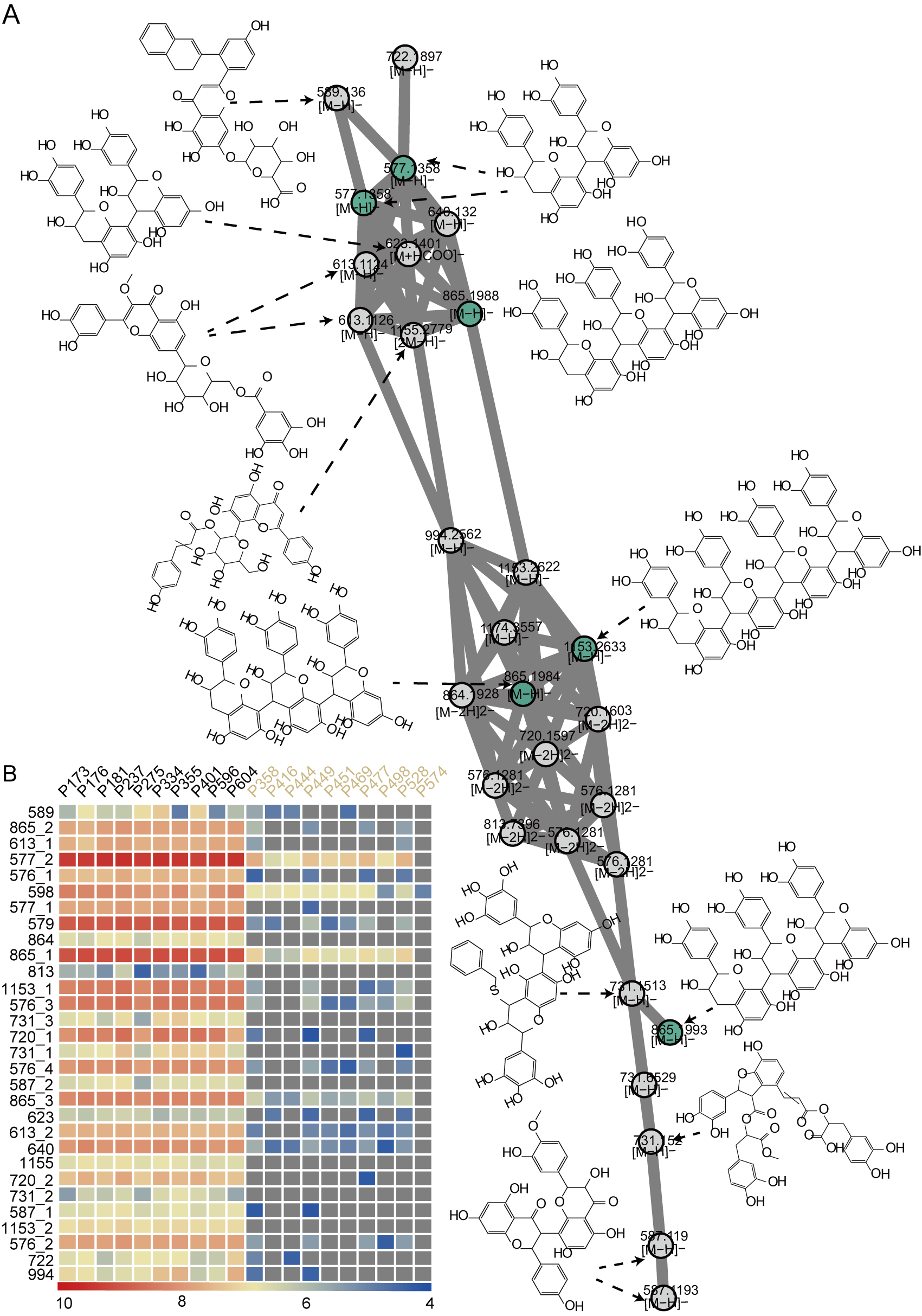

Supplement: Supplementary file 1 [file plants-14-00753-s001.zip › FigS3.tif]
